# St. Bartholomew's Hospital

**Published:** 1829-12

**Authors:** 


### ST. BARTHOLOMEW'S HOSPITAL.

### *Case of large Scrotal Hernia, reduced by general means.*

John Ross, ætat. twenty-eight, admitted September 17th, into Powell's ward, under Mr. Earle, with a large scrotal hernia of the right side. The man states that, two years since, he ruptured himself while lifting a heavy weight. He felt something to give way at the time, and observed a swelling in the groin, which gradually increased, and descended into the scrotum. For the space of about two years he suffered no inconvenience from the rupture, which was always protruded during the day, and returned again upon his going to rest at night. During this time he never wore a truss. About three weeks before his admission, he found that he was unable to return the hernia as usual. He states that, from this time, the swelling gradually increased in size, the scrotum being much more distended than he had ever observed it to be before; and its friction against the thighs causing him much uneasiness and pain, he was induced to seek relief at this hospital.

When admitted, the scrotum was found to be considerably distended with a hard and inelastic mass, which was supposed to consist of omentum; the testicle could be distinguished at the most inferior part of the scrotum; bowels not constipated. The protruded parts firmly resisted any attempts at reduction by the taxis; and, as there were no urgent symptoms, it was resolved to give the emaciating plan a fair trial, with the view of lessening the bulk of the tumor by reducing the quantity of fat, of which it was thought chiefly to consist. As Mr. Earle expressed it, he would try to convert his fat into *soap*, by the internal exhibition of the liquor potassæ.

The patient was put upon low diet, and the recumbent posture was strictly enjoined. He was ordered to take Liq. Potassæ ♏xx. ter die; and, with the view of assisting the absorbents, the Ung. Hydrargyri was directed to be rubbed in, so as to produce slight ptyalism. The good effects of the treatment were in a short time manifest, and at the end of five weeks the plan had succeeded perfectly. The hardened omentum gradually softened and decreased in bulk, until it was entirely reduced. The neck of the sac was felt to be considerably thickened.

October 24th, he was ordered to have a truss, and discharged on the 27th.

---

*Luxation of the Thigh-bone upwards and forwards on the Pubes.*

Mary Hinds, ætat. sixty-three, was admitted into this hospital, October 19th. She said that she slipped down stairs and fell on the right hip, with the leg under her. The dresser was not able to ascertain exactly the nature of the accident, but recognised the head of the bone, which obeyed the motions of the limb, in the groin. Mr. Vincent saw her on the 20th, and showed the case to Messrs. Lawrence and Earle, when it was decided to have a dislocation upwards.

On examining the limb, it appeared about two thirds of an inch shorter than the other; the toes everted and pointed downwards, so as not to allow the heel to reach the ground; the knee was also everted; the heel and knee could be brought close to the opposite limb; the femur was not bent either backwards or forwards with regard to the pelvis, and could easily be flexed to a right angle with the pelvis; *it could not be rotated inwards,* and but slightly outwards; by using a slight extension, the limb could nearly be brought down to its original length; the hip was flattened externally. Situation of the head of the femur, upon and rather to the pubic side of the inferior, anterior, spinous process of the ilium, having no muscle between it and that process; in front, and covering that part of the head nearest the neck, was the origin of the rectus muscle that arises from the inferior spine; externally and laterally, the origins of the sartorius and tensor vaginæ femoris, which crossed the neck; on the pubic side, and rather in front, were the iliacus internus and crural nerve; the psoæ muscles and vessels still more internally; above was the crural arch; the origin of the rectus appeared the chief obstacle to still greater shortening of the limb. There was no pain or numbness from the pressure on the nerve when the limb was at rest.

Reduction was first attempted without the assistance of pulleys, the woman lying on her back, and extension made in a line with the body; but, as this plan did not succeed, she was placed on the sound hip (the left), and the pulleys applied, using extension in a direction posterior to the body. Mr. Earle at the same time pressed down the head of the bone, by which means the reduction was effected in eight minutes.

The age of this woman caused suspicion of fracture of the neck of the bone. The limb was not in this case flexed either backwards, as mentioned by Boyer, or forwards, according to Sir A. Cooper's authority, by reason of the small size and weakness of the muscles of the patient. The only diagnoses between this accident and fracture of the neck, were protuberance in the groin obeying the motions of the limb, and inability to rotate the limb inwards.

Two similar accidents have occurred at the Manchester Infirmary within the four last years, the latter within ten months: in both of these the thigh was flexed on the pelvis. Another case was recently admitted into an hospital in Dublin.—*Med. Gazette.*

---

